# Supplementary figures and images for: Retinal microvascular and neuronal pathologies probed in vivo by adaptive optical two-photon fluorescence microscopy
Source: eLife. 2023 Apr 11;12:e84853. doi: 10.7554/eLife.84853 (PMC10089658; doi:10.7554/eLife.84853)

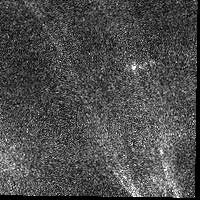

Supplement: Figure 1—source data 1. — ‘1-NoAO_8fAvg_stack.tif’ (No AO) and ‘2-AO_8fAvg_stack.tif’ (AO) Figure 1—source data 2 Source image stacks of retinal dendrites (Figure 1C): ‘1-NoAO_8fAvg_160_100_stack.tif’ (No AO) and ‘2-AO_8fAvg_160_100_stack.tif’ (AO). [file elife-84853-fig1-data1.zip › Figure1-source_data_1/1-NoAO_8fAvg_stack.tif]

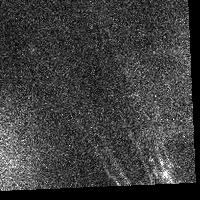

Supplement: Figure 1—source data 1. — ‘1-NoAO_8fAvg_stack.tif’ (No AO) and ‘2-AO_8fAvg_stack.tif’ (AO) Figure 1—source data 2 Source image stacks of retinal dendrites (Figure 1C): ‘1-NoAO_8fAvg_160_100_stack.tif’ (No AO) and ‘2-AO_8fAvg_160_100_stack.tif’ (AO). [file elife-84853-fig1-data1.zip › Figure1-source_data_1/2-AO_8fAvg_stack.tif]

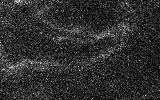

Supplement: Figure 1—source data 2. [file elife-84853-fig1-data2.zip › Figure1-source_data_2/1-NoAO_8fAvg_160_100_stack.tif]

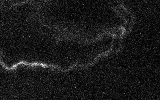

Supplement: Figure 1—source data 2. [file elife-84853-fig1-data2.zip › Figure1-source_data_2/2-AO_8fAvg_160_100_stack.tif]

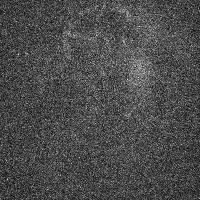

Supplement: Figure 2—source data 1. — ‘1-NoAO_8fAvg_stack.tif’ (No AO) and ‘2-AO_8fAvg_stack.tif’. [file elife-84853-fig2-data1.zip › Figure2-source_data_1/1-NoAO_8fAvg_stack.tif]

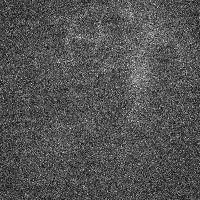

Supplement: Figure 2—source data 1. — ‘1-NoAO_8fAvg_stack.tif’ (No AO) and ‘2-AO_8fAvg_stack.tif’. [file elife-84853-fig2-data1.zip › Figure2-source_data_1/2-AO-8fAvg_stack.tif]

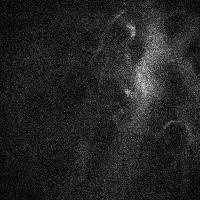

Supplement: Figure 2—source data 2. — ‘AO [1]’ stack: ‘1-AO[1]_Location_0_0_stack-9fAVG.tif’, ‘AO [2]’ stack: ‘2-AO[2]_Location_N15_N18_stack-9fAVG.tif’ ‘AO [3]’ stack: ‘3-AO[3]_Location_P4_P25_stack-9fAVG.tif’. [file elife-84853-fig2-data2.zip › Figure2-source_data_2/1-AO[1]_Location_0_0_stack-9fAVG.tif]

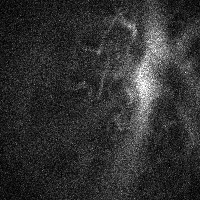

Supplement: Figure 2—source data 2. — ‘AO [1]’ stack: ‘1-AO[1]_Location_0_0_stack-9fAVG.tif’, ‘AO [2]’ stack: ‘2-AO[2]_Location_N15_N18_stack-9fAVG.tif’ ‘AO [3]’ stack: ‘3-AO[3]_Location_P4_P25_stack-9fAVG.tif’. [file elife-84853-fig2-data2.zip › Figure2-source_data_2/2-AO[2]_Location_N15_N18_stack-9fAVG.tif]

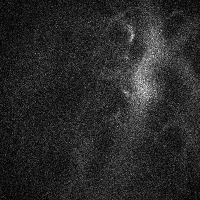

Supplement: Figure 2—source data 2. — ‘AO [1]’ stack: ‘1-AO[1]_Location_0_0_stack-9fAVG.tif’, ‘AO [2]’ stack: ‘2-AO[2]_Location_N15_N18_stack-9fAVG.tif’ ‘AO [3]’ stack: ‘3-AO[3]_Location_P4_P25_stack-9fAVG.tif’. [file elife-84853-fig2-data2.zip › Figure2-source_data_2/3-AO[3]_Location_P4_P25_stack-9fAVG.tif]

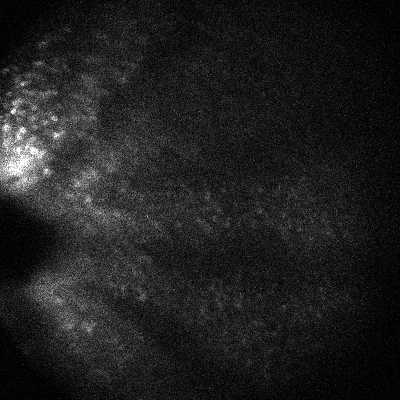

Supplement: Figure 3—source data 1. — ‘1-NoAO_60_84_8fAvg.tif’ (No AO) and ‘2-AO_60_84_8fAvg.tif’ (AO). [file elife-84853-fig3-data1.zip › Figure3-source_data_1/1-NoAO_60_84_8fAvg.tif]

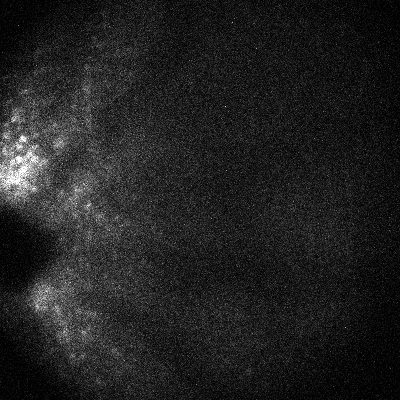

Supplement: Figure 3—source data 1. — ‘1-NoAO_60_84_8fAvg.tif’ (No AO) and ‘2-AO_60_84_8fAvg.tif’ (AO). [file elife-84853-fig3-data1.zip › Figure3-source_data_1/2-AO_60_84_8fAvg.tif]

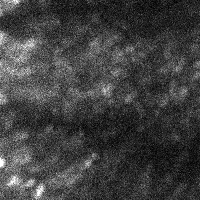

Supplement: Figure 3—source data 2. — ‘1-NoAO_8fAvg_stack.tif’ (No AO) and ‘2-AO_8fAvg_stack.tif’ (AO). [file elife-84853-fig3-data2.zip › Figure3-source_data_2/1-NoAO_8fAvg_stack.tif]

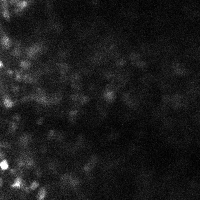

Supplement: Figure 3—source data 2. — ‘1-NoAO_8fAvg_stack.tif’ (No AO) and ‘2-AO_8fAvg_stack.tif’ (AO). [file elife-84853-fig3-data2.zip › Figure3-source_data_2/2-AO_8fAvg_stack.tif]

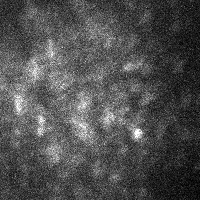

Supplement: Figure 3—source data 3. — ‘1-central_AO_20_28_8fAvg.tif’ (Central AO) and ‘2-local_AO_20_28_8fAvg.tif’ (Local AO). [file elife-84853-fig3-data3.zip › Figure3-source_data_3/1-central_AO_20_28_8fAvg.tif]

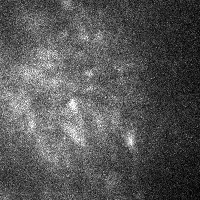

Supplement: Figure 3—source data 3. — ‘1-central_AO_20_28_8fAvg.tif’ (Central AO) and ‘2-local_AO_20_28_8fAvg.tif’ (Local AO). [file elife-84853-fig3-data3.zip › Figure3-source_data_3/2-local_AO_20_28_8fAvg.tif]

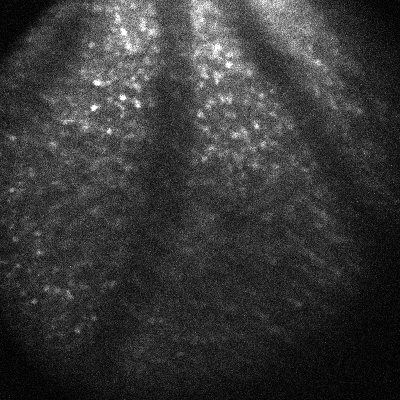

Supplement: Figure 4—source data 1. — ‘No AO’ stack: ‘1_NoAO_9fAvg_stack.tif’‘AO [i]’ stack: ‘2_AO[i]_2_2.8_9fAvg_stack.tif’ ‘AO [ii]’ stack: ‘3_AO[ii]_10_14_9fAvg_stack.tif’ ‘AO [iii]’ stack: ‘4_AO[iii]_20_28_9fAvg_stack.tif’ ‘AO [iv]’ stack: ‘5_AO[iv]_40_56_9fAvg_stack.tif’. [file elife-84853-fig4-data1.zip › Figure4-source_data_1/1_NoAO_9fAvg_stack.tif]

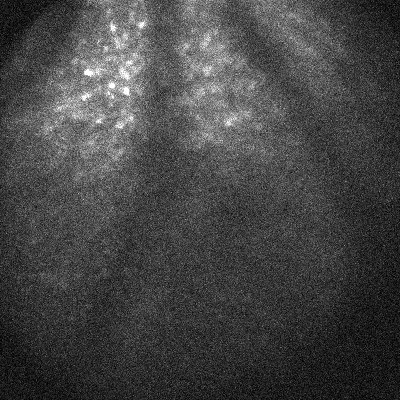

Supplement: Figure 4—source data 1. — ‘No AO’ stack: ‘1_NoAO_9fAvg_stack.tif’‘AO [i]’ stack: ‘2_AO[i]_2_2.8_9fAvg_stack.tif’ ‘AO [ii]’ stack: ‘3_AO[ii]_10_14_9fAvg_stack.tif’ ‘AO [iii]’ stack: ‘4_AO[iii]_20_28_9fAvg_stack.tif’ ‘AO [iv]’ stack: ‘5_AO[iv]_40_56_9fAvg_stack.tif’. [file elife-84853-fig4-data1.zip › Figure4-source_data_1/2_AO[i]_2_2.8_9fAvg_stack.tif]

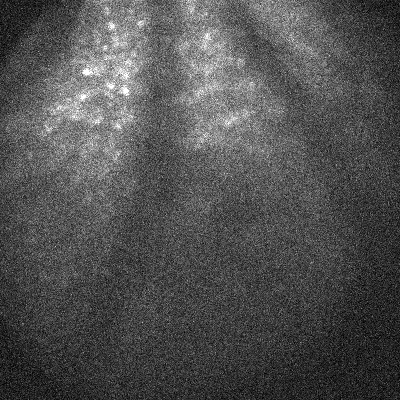

Supplement: Figure 4—source data 1. — ‘No AO’ stack: ‘1_NoAO_9fAvg_stack.tif’‘AO [i]’ stack: ‘2_AO[i]_2_2.8_9fAvg_stack.tif’ ‘AO [ii]’ stack: ‘3_AO[ii]_10_14_9fAvg_stack.tif’ ‘AO [iii]’ stack: ‘4_AO[iii]_20_28_9fAvg_stack.tif’ ‘AO [iv]’ stack: ‘5_AO[iv]_40_56_9fAvg_stack.tif’. [file elife-84853-fig4-data1.zip › Figure4-source_data_1/3_AO[ii]_10_14_9fAvg_stack.tif]

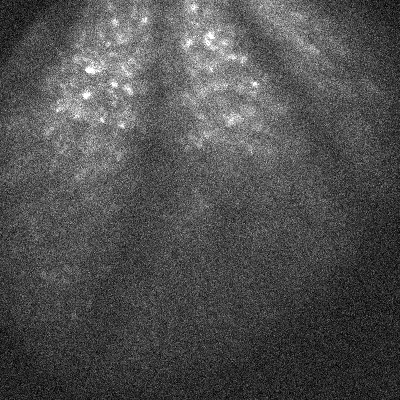

Supplement: Figure 4—source data 1. — ‘No AO’ stack: ‘1_NoAO_9fAvg_stack.tif’‘AO [i]’ stack: ‘2_AO[i]_2_2.8_9fAvg_stack.tif’ ‘AO [ii]’ stack: ‘3_AO[ii]_10_14_9fAvg_stack.tif’ ‘AO [iii]’ stack: ‘4_AO[iii]_20_28_9fAvg_stack.tif’ ‘AO [iv]’ stack: ‘5_AO[iv]_40_56_9fAvg_stack.tif’. [file elife-84853-fig4-data1.zip › Figure4-source_data_1/4_AO[iii]_20_28_9fAvg_stack.tif]

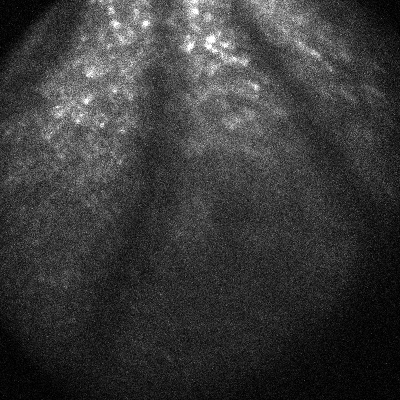

Supplement: Figure 4—source data 1. — ‘No AO’ stack: ‘1_NoAO_9fAvg_stack.tif’‘AO [i]’ stack: ‘2_AO[i]_2_2.8_9fAvg_stack.tif’ ‘AO [ii]’ stack: ‘3_AO[ii]_10_14_9fAvg_stack.tif’ ‘AO [iii]’ stack: ‘4_AO[iii]_20_28_9fAvg_stack.tif’ ‘AO [iv]’ stack: ‘5_AO[iv]_40_56_9fAvg_stack.tif’. [file elife-84853-fig4-data1.zip › Figure4-source_data_1/5_AO[iv]_40_56_9fAvg_stack.tif]

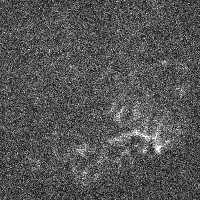

Supplement: Figure 5—source data 1. — ‘1-NoAO_8fAvg_60_84_stack.tif’ (No AO) and ‘2-AO_8fAvg_60_84_stack.tif’ (AO). Source image stacks of VLDLR-KO/Sca1-GFP mouse retina (Figure 5A, zoomed-in view). ‘3-NoAO_20_28_8fAvg_stack.tif’ (No AO) and ‘4-AO_20_28_8fAvg_stack.tif’ (AO). [file elife-84853-fig5-data1.zip › Figure5-source_data_1/1-NoAO_8fAvg_60_84_stack.tif]

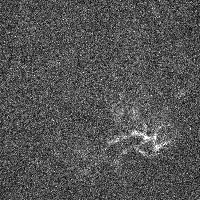

Supplement: Figure 5—source data 1. — ‘1-NoAO_8fAvg_60_84_stack.tif’ (No AO) and ‘2-AO_8fAvg_60_84_stack.tif’ (AO). Source image stacks of VLDLR-KO/Sca1-GFP mouse retina (Figure 5A, zoomed-in view). ‘3-NoAO_20_28_8fAvg_stack.tif’ (No AO) and ‘4-AO_20_28_8fAvg_stack.tif’ (AO). [file elife-84853-fig5-data1.zip › Figure5-source_data_1/2-AO_8fAvg_60_84_stack.tif]

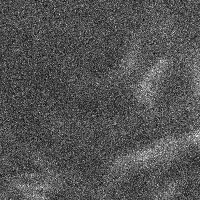

Supplement: Figure 5—source data 1. — ‘1-NoAO_8fAvg_60_84_stack.tif’ (No AO) and ‘2-AO_8fAvg_60_84_stack.tif’ (AO). Source image stacks of VLDLR-KO/Sca1-GFP mouse retina (Figure 5A, zoomed-in view). ‘3-NoAO_20_28_8fAvg_stack.tif’ (No AO) and ‘4-AO_20_28_8fAvg_stack.tif’ (AO). [file elife-84853-fig5-data1.zip › Figure5-source_data_1/3-NoAO_20_28_8fAvg_stack.tif]

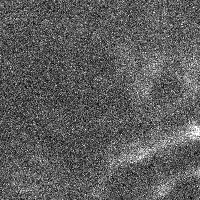

Supplement: Figure 5—source data 1. — ‘1-NoAO_8fAvg_60_84_stack.tif’ (No AO) and ‘2-AO_8fAvg_60_84_stack.tif’ (AO). Source image stacks of VLDLR-KO/Sca1-GFP mouse retina (Figure 5A, zoomed-in view). ‘3-NoAO_20_28_8fAvg_stack.tif’ (No AO) and ‘4-AO_20_28_8fAvg_stack.tif’ (AO). [file elife-84853-fig5-data1.zip › Figure5-source_data_1/4-AO_20_28_8fAvg_stack.tif]

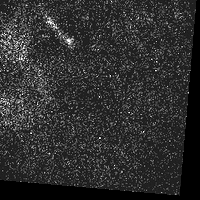

Supplement: Figure 5—source data 2. — ‘1-NoAO_60_84_Stack.tif’ (No AO) and ‘2-AO_60_84_Stack.tif’ (AO). Source image stacks of WT/Sca1-GFP mouse retina (Figure 5B, zoomed-in view). ‘3-NoAO_20_28_stack.tif’ (No AO) and ‘4-AO_reged_8fAvg_stack.tif’ (AO). [file elife-84853-fig5-data2.zip › Figure5-source_data_2/1-NoAO_60_84_Stack.tif]

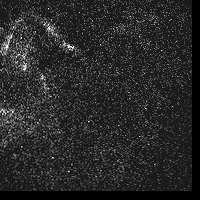

Supplement: Figure 5—source data 2. — ‘1-NoAO_60_84_Stack.tif’ (No AO) and ‘2-AO_60_84_Stack.tif’ (AO). Source image stacks of WT/Sca1-GFP mouse retina (Figure 5B, zoomed-in view). ‘3-NoAO_20_28_stack.tif’ (No AO) and ‘4-AO_reged_8fAvg_stack.tif’ (AO). [file elife-84853-fig5-data2.zip › Figure5-source_data_2/2-AO_60_84_Stack.tif]

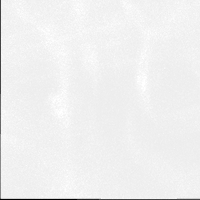

Supplement: Figure 5—source data 2. — ‘1-NoAO_60_84_Stack.tif’ (No AO) and ‘2-AO_60_84_Stack.tif’ (AO). Source image stacks of WT/Sca1-GFP mouse retina (Figure 5B, zoomed-in view). ‘3-NoAO_20_28_stack.tif’ (No AO) and ‘4-AO_reged_8fAvg_stack.tif’ (AO). [file elife-84853-fig5-data2.zip › Figure5-source_data_2/3-NoAO_20_28_stack.tif]

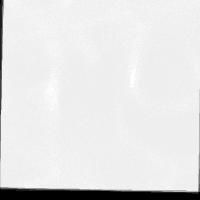

Supplement: Figure 5—source data 2. — ‘1-NoAO_60_84_Stack.tif’ (No AO) and ‘2-AO_60_84_Stack.tif’ (AO). Source image stacks of WT/Sca1-GFP mouse retina (Figure 5B, zoomed-in view). ‘3-NoAO_20_28_stack.tif’ (No AO) and ‘4-AO_reged_8fAvg_stack.tif’ (AO). [file elife-84853-fig5-data2.zip › Figure5-source_data_2/4-AO_reged_8fAvg_stack.tif]

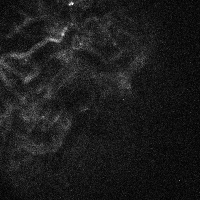

Supplement: Figure 5—source data 3. — ‘1-AO_8fAvg_stack.tif’. Source image stacks of VLDLR-KO/Sca1-GFP mouse retina (Figure 5C, zoomed-in view). ‘2-AO_zoomin_8fAvg_stack.tif’. [file elife-84853-fig5-data3.zip › Figure5-source_data_3/1-AO_8fAvg_stack.tif]

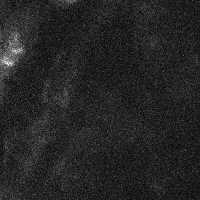

Supplement: Figure 5—source data 3. — ‘1-AO_8fAvg_stack.tif’. Source image stacks of VLDLR-KO/Sca1-GFP mouse retina (Figure 5C, zoomed-in view). ‘2-AO_zoomin_8fAvg_stack.tif’. [file elife-84853-fig5-data3.zip › Figure5-source_data_3/2-AO_zoomin_8fAvg_stack.tif]

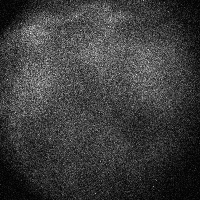

Supplement: Figure 5—source data 4. — ‘1-NoAO-8fAvg_stack.tif’. [file elife-84853-fig5-data4.zip › Figure5-source_data_4/1-NoAO-8fAvg_stack.tif]

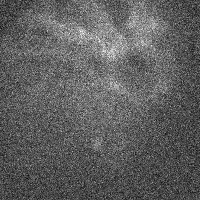

Supplement: Figure 5—source data 5. — ‘1-NoAO-8fAvg_stack.tif’. [file elife-84853-fig5-data5.zip › Figure5-source_data_5/1-NoAO-8fAvg_stack.tif]

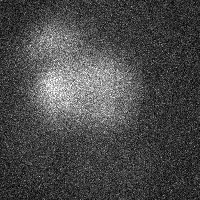

Supplement: Figure 5—source data 6. — Day 1: ‘1A-Day1-NIR_EB_8fAvg_stack.tif’ and ‘1B-Day1-GFP_60_84_8fAvg_stack.tif’ Day 2: ‘2A-Day2-NIR_EB_8fAvg_stack.tif’ and ‘2B-Day2-GFP_60_84_8fAvg_stack.tif’ Day 3: ‘3A-Day3-NIR_EB_8fAvg_stack.tif’ and ‘3B-Day3-GFP_60_84_8fAvg_stack.tif’ Source image stacks of microglia (Figure 5G). ‘4A-Day3-microglia_t=0.tif’, ‘4B-Day3-microglia_t=20.tif’, and ‘4C-Day3-microglia_t=40.tif’. [file elife-84853-fig5-data6.zip › Figure5-source_data_6/1A-Day1-NIR_EB_8fAvg_stack.tif]

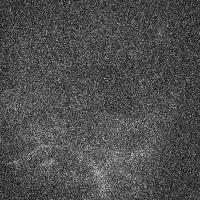

Supplement: Figure 5—source data 6. — Day 1: ‘1A-Day1-NIR_EB_8fAvg_stack.tif’ and ‘1B-Day1-GFP_60_84_8fAvg_stack.tif’ Day 2: ‘2A-Day2-NIR_EB_8fAvg_stack.tif’ and ‘2B-Day2-GFP_60_84_8fAvg_stack.tif’ Day 3: ‘3A-Day3-NIR_EB_8fAvg_stack.tif’ and ‘3B-Day3-GFP_60_84_8fAvg_stack.tif’ Source image stacks of microglia (Figure 5G). ‘4A-Day3-microglia_t=0.tif’, ‘4B-Day3-microglia_t=20.tif’, and ‘4C-Day3-microglia_t=40.tif’. [file elife-84853-fig5-data6.zip › Figure5-source_data_6/1B-Day1-GFP_60_84_8fAvg_stack.tif]

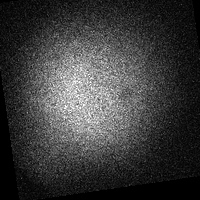

Supplement: Figure 5—source data 6. — Day 1: ‘1A-Day1-NIR_EB_8fAvg_stack.tif’ and ‘1B-Day1-GFP_60_84_8fAvg_stack.tif’ Day 2: ‘2A-Day2-NIR_EB_8fAvg_stack.tif’ and ‘2B-Day2-GFP_60_84_8fAvg_stack.tif’ Day 3: ‘3A-Day3-NIR_EB_8fAvg_stack.tif’ and ‘3B-Day3-GFP_60_84_8fAvg_stack.tif’ Source image stacks of microglia (Figure 5G). ‘4A-Day3-microglia_t=0.tif’, ‘4B-Day3-microglia_t=20.tif’, and ‘4C-Day3-microglia_t=40.tif’. [file elife-84853-fig5-data6.zip › Figure5-source_data_6/2A-Day2-NIR_EB_8fAvg_stack.tif]

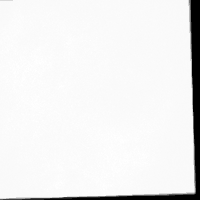

Supplement: Figure 5—source data 6. — Day 1: ‘1A-Day1-NIR_EB_8fAvg_stack.tif’ and ‘1B-Day1-GFP_60_84_8fAvg_stack.tif’ Day 2: ‘2A-Day2-NIR_EB_8fAvg_stack.tif’ and ‘2B-Day2-GFP_60_84_8fAvg_stack.tif’ Day 3: ‘3A-Day3-NIR_EB_8fAvg_stack.tif’ and ‘3B-Day3-GFP_60_84_8fAvg_stack.tif’ Source image stacks of microglia (Figure 5G). ‘4A-Day3-microglia_t=0.tif’, ‘4B-Day3-microglia_t=20.tif’, and ‘4C-Day3-microglia_t=40.tif’. [file elife-84853-fig5-data6.zip › Figure5-source_data_6/2B-Day2-GFP_60_84_8fAvg_stack.tif]

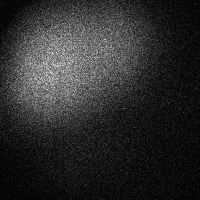

Supplement: Figure 5—source data 6. — Day 1: ‘1A-Day1-NIR_EB_8fAvg_stack.tif’ and ‘1B-Day1-GFP_60_84_8fAvg_stack.tif’ Day 2: ‘2A-Day2-NIR_EB_8fAvg_stack.tif’ and ‘2B-Day2-GFP_60_84_8fAvg_stack.tif’ Day 3: ‘3A-Day3-NIR_EB_8fAvg_stack.tif’ and ‘3B-Day3-GFP_60_84_8fAvg_stack.tif’ Source image stacks of microglia (Figure 5G). ‘4A-Day3-microglia_t=0.tif’, ‘4B-Day3-microglia_t=20.tif’, and ‘4C-Day3-microglia_t=40.tif’. [file elife-84853-fig5-data6.zip › Figure5-source_data_6/3A-Day3-NIR_EB_8fAvg_stack.tif]

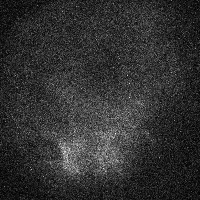

Supplement: Figure 5—source data 6. — Day 1: ‘1A-Day1-NIR_EB_8fAvg_stack.tif’ and ‘1B-Day1-GFP_60_84_8fAvg_stack.tif’ Day 2: ‘2A-Day2-NIR_EB_8fAvg_stack.tif’ and ‘2B-Day2-GFP_60_84_8fAvg_stack.tif’ Day 3: ‘3A-Day3-NIR_EB_8fAvg_stack.tif’ and ‘3B-Day3-GFP_60_84_8fAvg_stack.tif’ Source image stacks of microglia (Figure 5G). ‘4A-Day3-microglia_t=0.tif’, ‘4B-Day3-microglia_t=20.tif’, and ‘4C-Day3-microglia_t=40.tif’. [file elife-84853-fig5-data6.zip › Figure5-source_data_6/3B-Day3-GFP_60_84_8fAvg_stack.tif]

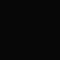

Supplement: Figure 5—source data 6. — Day 1: ‘1A-Day1-NIR_EB_8fAvg_stack.tif’ and ‘1B-Day1-GFP_60_84_8fAvg_stack.tif’ Day 2: ‘2A-Day2-NIR_EB_8fAvg_stack.tif’ and ‘2B-Day2-GFP_60_84_8fAvg_stack.tif’ Day 3: ‘3A-Day3-NIR_EB_8fAvg_stack.tif’ and ‘3B-Day3-GFP_60_84_8fAvg_stack.tif’ Source image stacks of microglia (Figure 5G). ‘4A-Day3-microglia_t=0.tif’, ‘4B-Day3-microglia_t=20.tif’, and ‘4C-Day3-microglia_t=40.tif’. [file elife-84853-fig5-data6.zip › Figure5-source_data_6/4A-Day3-microglia_t=0.tif]

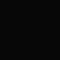

Supplement: Figure 5—source data 6. — Day 1: ‘1A-Day1-NIR_EB_8fAvg_stack.tif’ and ‘1B-Day1-GFP_60_84_8fAvg_stack.tif’ Day 2: ‘2A-Day2-NIR_EB_8fAvg_stack.tif’ and ‘2B-Day2-GFP_60_84_8fAvg_stack.tif’ Day 3: ‘3A-Day3-NIR_EB_8fAvg_stack.tif’ and ‘3B-Day3-GFP_60_84_8fAvg_stack.tif’ Source image stacks of microglia (Figure 5G). ‘4A-Day3-microglia_t=0.tif’, ‘4B-Day3-microglia_t=20.tif’, and ‘4C-Day3-microglia_t=40.tif’. [file elife-84853-fig5-data6.zip › Figure5-source_data_6/4B-Day3-microglia_t=20.tif]

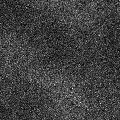

Supplement: Figure 5—source data 6. — Day 1: ‘1A-Day1-NIR_EB_8fAvg_stack.tif’ and ‘1B-Day1-GFP_60_84_8fAvg_stack.tif’ Day 2: ‘2A-Day2-NIR_EB_8fAvg_stack.tif’ and ‘2B-Day2-GFP_60_84_8fAvg_stack.tif’ Day 3: ‘3A-Day3-NIR_EB_8fAvg_stack.tif’ and ‘3B-Day3-GFP_60_84_8fAvg_stack.tif’ Source image stacks of microglia (Figure 5G). ‘4A-Day3-microglia_t=0.tif’, ‘4B-Day3-microglia_t=20.tif’, and ‘4C-Day3-microglia_t=40.tif’. [file elife-84853-fig5-data6.zip › Figure5-source_data_6/4C-Day3-microglia_t=40.tif]

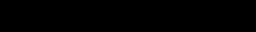

Supplement: Figure 6—source data 1. — ‘1A-RGC1_before-and-during_lidocaine.tiff’ and ‘1A-RGC1_before-and-during_lidocaine.mat’ ‘1B-RGC1_lidocaine-washout.tiff’ and ‘1B-RGC1_lidocaine-washout.mat’ RGC #2. ‘2A-RGC2_before-and-during_lidocaine.tiff’ and ‘2A-RGC2_lidocaine-washout.mat’ ‘2B-RGC2_lidocaine-washout.tiff’ and ‘2B-RGC2_lidocaine-washout.mat’. [file elife-84853-fig6-data1.zip › Figure6-source_data_1/1A-RGC1_before-and-during_lidocaine.tiff]

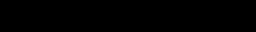

Supplement: Figure 6—source data 1. — ‘1A-RGC1_before-and-during_lidocaine.tiff’ and ‘1A-RGC1_before-and-during_lidocaine.mat’ ‘1B-RGC1_lidocaine-washout.tiff’ and ‘1B-RGC1_lidocaine-washout.mat’ RGC #2. ‘2A-RGC2_before-and-during_lidocaine.tiff’ and ‘2A-RGC2_lidocaine-washout.mat’ ‘2B-RGC2_lidocaine-washout.tiff’ and ‘2B-RGC2_lidocaine-washout.mat’. [file elife-84853-fig6-data1.zip › Figure6-source_data_1/1B-RGC1_lidocaine-washout.tiff]

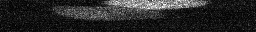

Supplement: Figure 6—source data 1. — ‘1A-RGC1_before-and-during_lidocaine.tiff’ and ‘1A-RGC1_before-and-during_lidocaine.mat’ ‘1B-RGC1_lidocaine-washout.tiff’ and ‘1B-RGC1_lidocaine-washout.mat’ RGC #2. ‘2A-RGC2_before-and-during_lidocaine.tiff’ and ‘2A-RGC2_lidocaine-washout.mat’ ‘2B-RGC2_lidocaine-washout.tiff’ and ‘2B-RGC2_lidocaine-washout.mat’. [file elife-84853-fig6-data1.zip › Figure6-source_data_1/2A-RGC2_before-and-during_lidocaine.tiff]

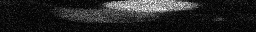

Supplement: Figure 6—source data 1. — ‘1A-RGC1_before-and-during_lidocaine.tiff’ and ‘1A-RGC1_before-and-during_lidocaine.mat’ ‘1B-RGC1_lidocaine-washout.tiff’ and ‘1B-RGC1_lidocaine-washout.mat’ RGC #2. ‘2A-RGC2_before-and-during_lidocaine.tiff’ and ‘2A-RGC2_lidocaine-washout.mat’ ‘2B-RGC2_lidocaine-washout.tiff’ and ‘2B-RGC2_lidocaine-washout.mat’. [file elife-84853-fig6-data1.zip › Figure6-source_data_1/2B-RGC2_lidocaine-washout.tiff]

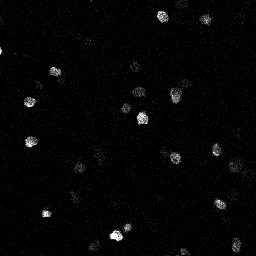

Supplement: Figure 6—source data 2. — ‘1-Sequence-i.tif’, ‘2-Sequence-ii.tif’, and ‘3-Sequence-iii.tif’. [file elife-84853-fig6-data2.zip › Figure6-source_data_2/1-Sequence-i.tif]

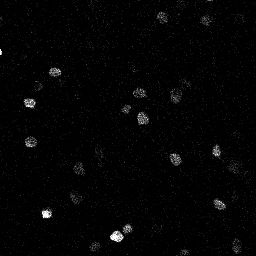

Supplement: Figure 6—source data 2. — ‘1-Sequence-i.tif’, ‘2-Sequence-ii.tif’, and ‘3-Sequence-iii.tif’. [file elife-84853-fig6-data2.zip › Figure6-source_data_2/2-Sequence-ii.tif]

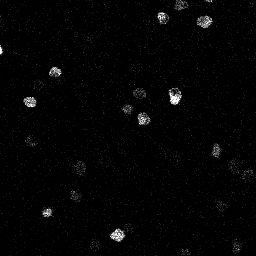

Supplement: Figure 6—source data 2. — ‘1-Sequence-i.tif’, ‘2-Sequence-ii.tif’, and ‘3-Sequence-iii.tif’. [file elife-84853-fig6-data2.zip › Figure6-source_data_2/3-Sequence-iIIi.tif]

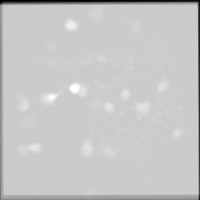

Supplement: Figure 6—source data 3. — ‘1-NoAO-large_FOV.tif’ (No AO) and ‘2-AO-large_FOV.tif’ (AO). Source images of rd1-Thy1-GCaMP6s mouse retina (Figure 6C, zoomed-in view). ‘3-NoAO-inset.tif’ (No AO) and ‘4-AO-inset.tif’ (AO). [file elife-84853-fig6-data3.zip › Figure6-source_data_3/2-AO-large_FOV.tif]

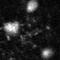

Supplement: Figure 6—source data 3. — ‘1-NoAO-large_FOV.tif’ (No AO) and ‘2-AO-large_FOV.tif’ (AO). Source images of rd1-Thy1-GCaMP6s mouse retina (Figure 6C, zoomed-in view). ‘3-NoAO-inset.tif’ (No AO) and ‘4-AO-inset.tif’ (AO). [file elife-84853-fig6-data3.zip › Figure6-source_data_3/4-AO-inset.tif]
